# National attitudes of medical students towards mandating the COVID-19 vaccine and its association with knowledge of the vaccine

**DOI:** 10.1371/journal.pone.0260898

**Published:** 2021-12-22

**Authors:** Danel Mayan, Kenny Nguyen, Brian Keisler

**Affiliations:** 1 University of South Carolina School of Medicine, Columbia, South Carolina, United States of America; 2 Department of Family and Preventive Medicine, University of South Carolina School of Medicine, Columbia, South Carolina, United States of America; Harper University Hospital, UNITED STATES

## Abstract

**Background:**

With the introduction of the novel COVID-19 vaccine, public hesitancy is being experienced with many turning to healthcare professionals for advice. As future physicians, medical students play a critical role in the public’s view of the vaccine.

**Objectives:**

To determine the attitude of U.S. medical students toward mandating the COVID-19 vaccine to healthcare workers and patients, as well as whether their knowledge of the vaccine plays a role in their view.

**Methods:**

The authors emailed a survey link to all U.S. medical schools with request to distribute it to their medical students. The survey remained open from 02/09/2021 to 03/15/2021 and included questions to determine the attitude of the medical students toward recommending the COVID-19 vaccine, and general knowledge questions about the vaccine. Chi square, Fisher’s exact test, and linear regression were conducted to determine associations between willingness to recommend the COVID-19 vaccine and general knowledge of the vaccine.

**Results:**

Among the 1,899 responses from medical students representing 151 U.S. medical schools, 57.82% approved of making the COVID-19 vaccine mandatory to healthcare workers, and 16.27% approved of making it mandatory to patients. Additionally, those who tested most knowledgeable of the vaccine were less likely to approve of making the vaccine mandatory for patients (66.67% vs. 72.70). Those that tested most knowledgeable were also more likely to personally receive the vaccine (72.35% vs 62.99%) as opposed to those that tested the least knowledgeable who were less willing to personally receive the vaccine (4.12% vs 14.17%).

**Conclusions:**

The data revealed that a slight majority of medical students support a vaccine mandate toward healthcare workers while a minority of medical students support a vaccine mandate toward healthcare workers. Additionally, medical students that had relatively high knowledge of the vaccine correlated with not approving of making the vaccine mandatory for patients. However increased knowledge of the vaccine correlated with increased willingness to personally receive the vaccine.

## Introduction

Vaccines against highly transmittable infectious diseases are one of the most effective medical interventions available and save millions of lives each year [1]. Previously, vaccines for infectious diseases required years of development and clinical evaluation before being authorized for use for the general population. Through recent advancements in rapid vaccine development and the unprecedented pressure placed on our healthcare systems and economies due to COVID-19, several vaccines for SARS-CoV-2, such as those manufactured by Pfizer BioNTech, Moderna, and Johnson & Johnson, have been approved for use by the Food and Drug Administration’s Emergency Use Authorization, with Pfizer BioNTech most recently receiving full FDA approval [2,3].

COVID-19 vaccine availability, however, does not guarantee uptake by healthcare workers [4]. Vaccine hesitancy–the delay of acceptance or refusal of vaccines despite the availability of vaccine services–remains a limiting factor for achieving optimal herd immunity against highly transmittable diseases. Refusal of vaccines is associated with an increased risk of contracting the target disease [5]. Several factors that drive vaccine hesitancy for the COVID-19 vaccine have been identified. Safety, quality control, side effects, and efficacy are among the most notable drivers of vaccine hesitancy for the COVID-19 vaccine, partly due to concerns related to the speed of the vaccine development and testing [6,7].

Although the vaccine has been proven to be effective at mitigating the effects of COVID-19, the hesitance of much of the public to receive the vaccine greatly limits the success of acquiring herd immunity. With these thoughts, the idea of a vaccine mandate has been brought up in the media especially after full FDA approval of the vaccine has been granted. Many businesses have already began requiring their employees to receive the vaccine, and more recently federal plans have been unveiled to mandate that employees of large business receive the vaccine or be subjected to weekly testing. Although controversial, some feel that a mandate is the only way to eradicate the disease.

Despite the potential reluctance and hesitancy of the public to accept any vaccine (e.g., influenza), health care providers remain trusted advisors and influencers of vaccination decisions. As an integral part of the healthcare team and as future physicians, medical students play a notable role in counseling patients about the novel coronavirus and providing accurate information about vaccination. Medical students will likely encounter vaccine-hesitant patients during their clinical rotations. Therefore, it is essential that medical students possess accurate knowledge of SARS-CoV-2 and the available vaccine options to dispel misinformation surrounding the COVID-19 vaccine. Previous studies have shown that medical students view immunization favorably and are more likely to gain comfort counseling vaccine-hesitant patients following a targeted curriculum on infectious disease and immunology [8].

This study was conducted to determine the attitudes of U.S. medical students toward receiving the COVID-19 vaccine in addition to their approval toward mandating the vaccine to patients and/or healthcare workers. Additionally, it also sought to determine whether their degree of knowledge about the vaccine plays a role in their view.

## Methods

**Fig 1:**
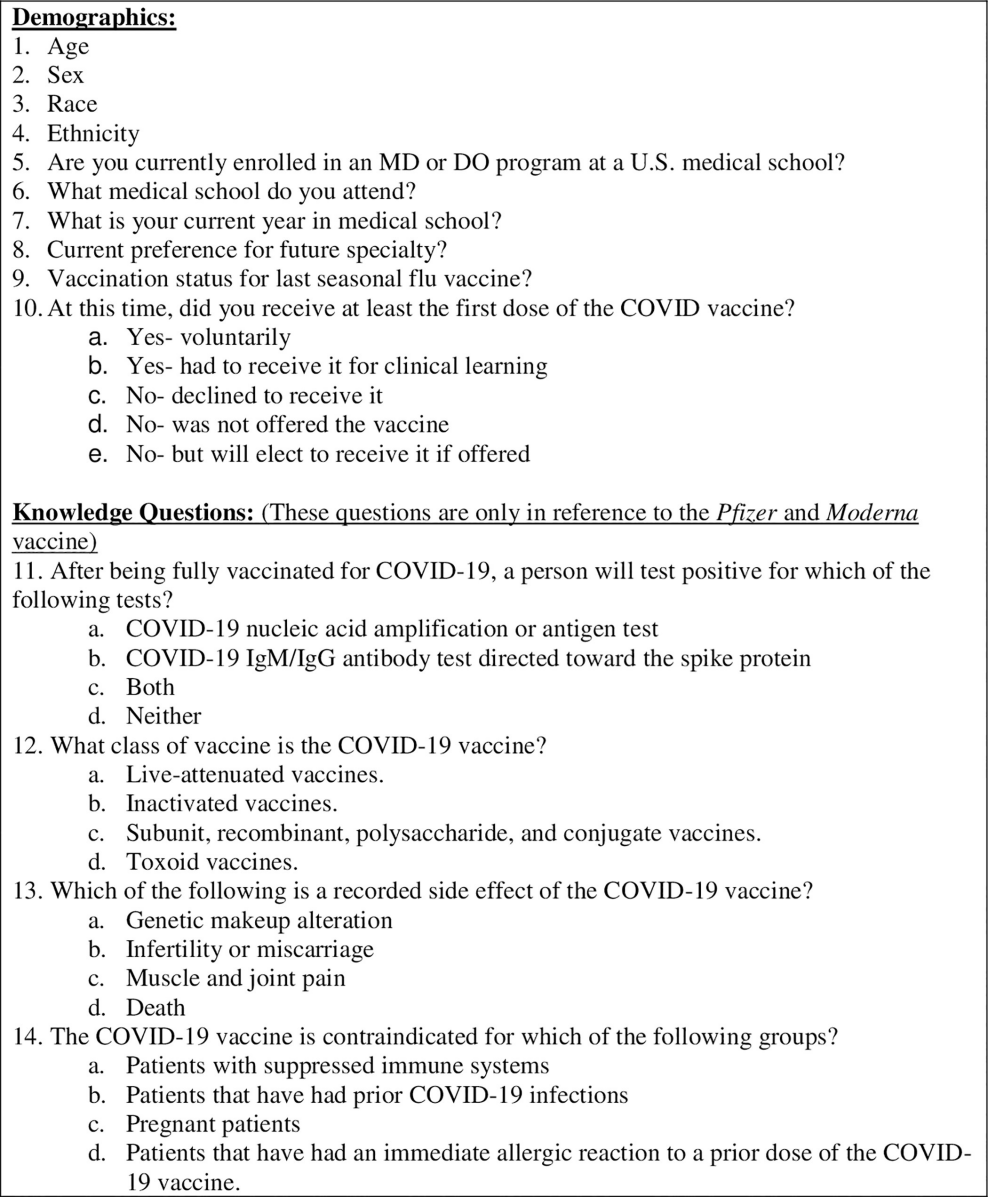
COVID-19 vaccination survey.

Data was collected with a survey created in REDCap, a secure, cloud-based survey and database software provided by the University of South Carolina. We conducted this study with a population of U.S. medical students. Inclusion criteria included being enrolled in a MD or DO program at a U.S. medical school. Participation was acquired via an email sent to the student affairs offices of all 212 U.S. medical schools with request to distribute the website link for the online survey (Fig 1) to their students. Follow up emails were sent to the research departments of schools that did not respond. Additional participation was recruited via medical student- targeted social media. Financial incentive was not provided for completion of the survey. A total of 2,025 responses were received. Among them, 126 students did not provide their demographic information, leaving 1,899 with full records.

The survey determined demographics (age, gender, race/ethnicity, and school year), assessed general knowledge about the Pfizer and Moderna COVID-19 vaccines, and attitudes toward vaccine recommendation/mandate practices. It remained open from 02/09/2021 to 03/15/2021. Contact information was not collected from participants and amount of survey clicks versus amount of survey completions was not recorded.

The surveyed medical students were asked to rank their knowledge of the COVID-19 vaccine on a scale of 1–3, with 3 being the most knowledgeable. This ranking was dichotomized as most knowledgeable (scale of 3) or not (scale of 1, 2). 5 knowledge questions about the COVID-19 vaccine were further included in the survey. The number of correctly answered questions was categorized into three levels: correctly answered 0–3 questions (the reference), correctly answered 4 questions, and correctly answered all 5 questions.

Three primary attitude questions were provided in the survey, including recommendation for the COVID-19 vaccine toward patients, recommendation for the COVID-19 vaccine toward healthcare workers, and attitude toward personally receiving the vaccine. Although the vaccine recommendations were originally designed as 3-level questions (“I would make it mandatory,” “I would recommend it,” and “I would not recommend it”), the “I would not recommend” option had too few respondents to allow for proper evaluation of statistics so the groups were dichotomized to those that would make the vaccine mandatory (“I would make it mandatory”) and those that would not make the vaccine mandatory (“I would recommend it” combined with “I would not recommend it”). The logic behind this grouping being that if a participant selected that they would not recommend the vaccine, it was also assumed that they would not recommend mandating it as well. Likewise, if a participant selected “I would recommend it” over “I would make it mandatory,” it was assumed that they did not support a mandate even though they still support recommending the vaccine, thus fitting into the “I would not make it mandatory” category. The 5-level attitude toward personally receiving the vaccine was also dichotomized, due to the same issue, as “will receive vaccine” (“willing” combined with “very willing”) or “will not receive the vaccine” (“indifferent” combined with “reluctant” and “very reluctant”).

Participants were given the option of leaving comments to explain or support their survey responses. These comments were grouped by similarity of content and quantified to determine the most commonly reported statements supporting or not supporting their survey selections.

Descriptive analysis was conducted to summarize the characteristics of the entire sample. Comparisons of demographics and knowledge were conducted by each of the four attitude outcomes, chi-square test for frequency and *t*-test for continuous variables. Additionally, multiple logistic regressions were used to estimate the odds ratio (OR) and 95% confidence interval (CI) of demographics and knowledge for each of the four attitude outcomes. All statistical analysis was conducted at the significance level of 0.95, using the software SAS version 9.4 (SAS Institute, Cary, North Carolina).

For this cross-sectional study, the University of South Carolina institutional review board designated this study as human subjects exempt, February 8, 2021. Informed consent was provided on the first page of the survey and respondents were made aware that by volunteering to complete and submit the survey they agreed to participate.

## Results

The sample size totaled 1899 medical students enrolled at an MD or DO program at a U.S. medical school. The average age of the study sample was 26 years old, with 64% being female. The detailed characteristics of the respondents are shown in Table 1. Although only 18% of medical students ranked themselves as being the most knowledgeable of the vaccine, the majority of surveyed medical students (71%) answered all 5 questions correctly, with less than 5% correctly answering only 0–3 questions. A minority of medical students (16%) designated their approval in making the vaccine mandatory for patients, while a slight majority (58%) designated their approval in making it mandatory for healthcare workers. Nearly all of the surveyed medical students (93%) designated that they would receive/ have received the vaccine willingly.

**Table 1:** Surveyed medical students’ demographics, knowledge, and attitude toward the COVID-19 vaccine.

Variables	Number	Percentage
Demographics		
Age (mean: years)	1899	25.79
<25 Years	649	34.18
25–29 years	1091	57.45
30 and over	159	8.37
Female	1221	64.30
Race and ethnicity		
Non-Hispanic White	1338	70.46
Minority (African American and Asian)	377	19.85
Other or Unknown	184	9.69
School Year		
1st year	578	30.44
2nd year	422	22.22
3rd year	450	23.70
4th year	449	23.64
Knowledge on COVID-19 vaccine		
Self-ranked as the most knowledgeable	341	17.96
Correctly answered 0–3 questions	91	4.79
Correctly answered 4 questions	446	23.49
Correctly answered 5 questions	1362	71.72
Attitude		
Make wearing face masks mandatory	1458	76.78
Make patient receiving COVID-19 vaccine mandatory	309	16.27
Make healthcare worker receiving COVID-19 vaccine mandatory	1096	57.71
Would willingly receive or did willingly receive the COVID-19 vaccine	1772	93.31

Medical students in the “would not recommend the vaccine” and “would not make the vaccine mandatory” group were combined into the “would not make the vaccine mandatory” group and compared to the “would make the vaccine mandatory.” These results are shown in Table 2. 77% of medical students selected that they would make wearing face masks mandatory, 16% approve of making the vaccine mandatory for patients, and 58% approve of making the vaccine mandatory for healthcare workers.

**Table 2:** Proportion of demographics and knowledge of the COVID-19 vaccine by attitude outcomes.

Variables (percentage)	Mask mandatory		Vaccine mandatory for patients		Vaccine mandatory for healthcare workers		Will receive vaccine	
	Yes	No	Yes	No	Yes	No	Yes	No
N	1458	441	309	1590	1096	803	1772	127
Demographics								
Age (mean: Years)	25.77	25.88	25.98	25.76	25.77	25.82	25.78	25.99
<25 Years	34.77	32.20	31.72	34.65	35.86	31.88	34.31	32.28
25–29 years	56.58	60.32	58.58	57.23	55.38*	60.27	57.45	57.48
30 and over	8.64	7.48	9.71	8.11	8.76	7.85	8.24	10.24
Female	67.70***	53.06	58.90*	65.35	63.50	65.38	65.18**	51.97
Race and ethnicity								
Non-Hispanic White	67.35***	80.73	60.19***	72.45	67.70**	74.22	70.26	73.23
Minority (African American and Asian)	22.57***	10.88	28.16***	18.24	22.81***	15.82	20.37*	12.60
Other or Unknown	10.08	8.39	11.65	9.31	9.49	9.96	9.37	14.17
School Year								
1st year	29.56	33.33	29.13	30.69	30.47	30.39	30.08	35.43
2nd year	23.87**	16.78	23.62	21.95	24.36**	19.30	22.18	22.83
3rd year	22.84	26.53	22.01	24.03	21.35**	26.90	23.53	25.98
4th year	23.73	23.36	25.24	23.33	23.81	23.41	24.21*	15.75
Knowledge on COVID-19 vaccine								
Self-ranked as the most knowledgeable	18.31	16.78	19.42	17.67	18.80	16.81	18.28	13.39
Correctly answered 0–3 questions	4.46	5.90	5.83	4.59	4.29	5.48	4.12***	14.17
Correctly answered 4 questions	23.46	23.58	27.51	22.70	24.18	22.54	23.53	22.83
Correctly answered 5 questions	72.09	70.52	66.67*	72.70	71.53	71.98	72.35*	62.99

*: p<0.05

**: p< 0.001

***: p< 0.0001.

Table 2 also displays the statistics of both demographics and knowledge on the COVID-19 vaccine when compared with attitude outcomes. The 25–29 year age group was less likely to make receiving the COVID-19 vaccine mandatory for healthcare workers (55.38% vs. 50.27%, p = 0.03), with the remaining ages not significantly associated with other attitude outcomes. Females were more likely to make wearing face mask mandatory (67.70% vs. 53.06%, p<0.0001) and would personally be willing to receive the vaccine (65.18% vs. 51.97%, p = 0.003), but were less likely to approve of making the vaccine mandatory for patients (58.90% vs. 65.35%, p = 0.03). Non-Hispanic Whites were less likely to make wearing face masks mandatory (67.35% vs. 80.73%, p<0.0001), less likely to approve of making the vaccine mandatory for patients (60.19% vs. 72.45%, p<0.0001) and for healthcare workers (67.70% vs. 74.22%, p<0.0001). African Americans and Asians were more likely to approve mandating wearing face masks (22.57% vs. 10.88%, p<0.0001), more likely approve of making the vaccine mandatory for patients (28.16% vs. 18.24%, p<0.0001) and healthcare workers (22.81% vs. 15.82%, p = 0.0002), and more likely to willingly receive the vaccine (20.37% vs. 12.60%, p = 0.03). Those who correctly answered 5 questions were less likely to approve of making the vaccine mandatory for patients (66.67% vs. 72.70%, p = 0.03) but were more likely to receive vaccine for themselves (72.35% vs 62.99%, p = 0.02). Those who correctly answered 0–3 questions were less willing to personally receive the vaccine (4.12% vs 14.17%, p<0.0001).

The association of demographics and knowledge of the COVID-19 vaccine with attitude outcomes were further examined with multiple logistic regression. Odds ratios and 95% confidence intervals were reported in Table 3. Females remained statistically significant: they were more likely to make wearing face masks mandatory (OR: 1.92, 95% CI: 1.53–2.39) and were more likely to willingly receive the vaccine (OR: 1.74, 95% CI: 1.20–2.52) than males. Compared with non-Hispanic Whites, African Americans and Asians were more likely to make wearing face masks mandatory (OR: 2.60, 95% CI: 1.87–3.62), more likely to make receiving the vaccine mandatory for patients (OR: 1.88, 95% CI: 1.40–2.51) as well as for healthcare workers (OR: 1.61, 95% CI: 1.27–2.05), and more willing to personally receive the vaccine (OR: 1.83, 95% CI: 1.06–3.18). Other or unknown race/ethnicity were more likely to make wearing face mask mandatory (OR: 1.47, 95% CI: 1.00–2.17), compared with non-Hispanic Whites. Compared with medical students in their first year of study, those in their second year were more likely to make wearing face masks mandatory (OR: 1.68, 95% CI: 1.21–2.32). Compared with those that correctly answered 0–3 questions correctly, medical students who correctly answered 4 questions correctly (OR: 3.30, 95% CI: 1.71–6.36) or 5 (OR: 3.57, 95% CI: 1.98–6.44) were more likely to personally receive the vaccine.

**Table 3:** Odds ratios and 95% confident intervals from the multiple regression of attitude on demographics and knowledge on COVID-19 vaccine.

Variables	Mask mandatory	Vaccine mandatory^1^	Vaccine mandatory^2^	Will receive vaccine
Demographics				
Age (Years)	1.00 (0.96, 1.04)	1.02 (0.98, 1.07)	1.00 (0.97, 1.04)	0.97 (0.92, 1.03)
Female	1.92 (1.53, 2.39)	0.78 (0.60, 1.01)	0.93 (0.77, 1.13)	1.74 (1.20, 2.52)
Race and ethnicity				
Non-Hispanic White	Ref	Ref	Ref	Ref
Minority (African American and Asian)	2.60 (1.87, 3.62)	1.88 (1.40, 2.51)	1.61 (1.27, 2.05)	1.83 (1.06, 3.18)
Other or Unknown	1.47 (1.00, 2.17)	1.49 (0.999, 2.22)	1.06 (0.78, 1.45)	0.71 (0.41, 1.22)
School Year				
1st year	Ref	Ref	Ref	Ref
2nd year	1.68 (1.21, 2.32)	1.16 (0.82, 1.63)	1.25 (0.96, 1.62)	1.05 (0.63, 1.73)
3rd year	0.97 (0.72, 1.30)	0.97 (0.68, 1.38)	0.78 (0.60, 1.01)	0.97 (0.59, 1.58)
4th year	1.23 (0.89, 1.69)	1.12 (0.78, 1.60)	1.00 (0.76, 1.31)	1.74 (0.98, 3.11)
Knowledge on COVID-19 vaccine				
Self-ranked as the most knowledgeable	1.28 (0.95, 1.71)	1.18 (0.86, 1.63)	1.20 (0.94, 1.53)	1.54 (0.90, 2.63)
Correctly answered 0–3 questions	Ref	Ref	Ref	Ref
Correctly answered 4 questions	1.22 (0.72, 2.06)	0.98 (0.55, 1.75)	1.43 (0.91, 2.27)	3.30 (1.71, 6.36)
Correctly answered 5 questions	1.28 (0.78, 2.10)	0.77 (0.44, 1.33)	1.34 (0.87, 2.07)	3.57 (1.98, 6.44)

Note:

1, Vaccine mandatory for patients

2, vaccine mandatory for healthcare workers.

## Discussion

This study was conducted to determine the attitudes of U.S. medical students toward receiving the COVID-19 vaccine in addition to their approval toward mandating the vaccine to patients and/or healthcare workers. Additionally, it also sought to determine whether their degree of knowledge about the vaccine plays a role in their view. Based on the correctly answered knowledge questions, participants were divided into 3 separate groups with each signifying a different level of knowledge of the vaccine. Those who scored 0–3 questions correctly were inferred to know little about the vaccine, those with 4 correctly answered questions were inferred to know a lot about the vaccine, and those with 5 correctly answers questions were inferred to having very good knowledge of the vaccine. However, only 0.95% of participants selected that they would not recommend the vaccine to patients, and 1.18% selected that they would not recommend the vaccine to healthcare workers, which limited our statistical analysis. To combat this, the “I would not recommend” and the “I would recommend” group were combined into a “I would not make it mandatory” group and compared to those that specifically selected that they would make the vaccine mandatory. Although slight differences between group data existed in terms of the amount of correctly answered question and their approval of making the vaccine mandatory, the only truly statistically significant result was that those who correctly answered all 5 questions were less likely to approve of making the vaccine mandatory for patients, however they were more likely to personally receive the vaccine. Additionally, those who were least knowledgeable of the vaccine with only 0–3 correctly answered questions were less willing to personally receive the vaccine. Therefore, these results suggest that a positive correlation exists between knowledge of the vaccine and willingness to personally receive it.

An additional aim of this study was to determine the overall willingness of medical students to recommend the vaccine. Among the surveyed students, 1.16% denoted themselves not being willing to recommend the vaccine to patients, and 0.95% were not willing to recommend it to healthcare workers. Additionally, only 0.58% of medical students showed any reluctance toward personally receiving the vaccine. This is in contrast to a similar study conducted at a single allopathic medical school that found that nearly a quarter of their medical students were hesitant about receiving the vaccine [9].

Comments submitted by participants were grouped based on similarity of content and were quantified to assess the most shared ideologies. Participants that indicated willingness to recommend the vaccine as well as toward personally receiving it included the following as factors that influenced their decision: FDA and CDC endorsement, data from clinical trials, wanting personal safety during patient exposure, desire to protect others, as well as eagerness to resolve the pandemic. Additionally, multiple medical students that indicated their approval toward willingness to recommend the vaccine yet indicated hesitancy to personally receiving it specified that their reasoning was that they did not want to take the opportunity away from people that were more at risk of complications from COVID-19.

Among the participants that indicated that they would not recommend the vaccine, the most reported factors that influenced their decision included lack of long-term effects of the vaccine, perceived lack of effectiveness, as well as speed of vaccine development.

Although the participant groups were dichotomized, a limitation to this study was the relatively small proportion of medical students selecting unwillingness to recommend the vaccine in relation to those willing to recommend it. This prompted us to alter the scope of the study to question attitudes toward vaccine mandates as opposed to primarily focusing on vaccine recommendation. An additional limitation is that the medical students’ opinions regarding making the COVID vaccine mandatory may change over time, especially as more data is made available.

Our results were able to show an association between high knowledge of the COVID vaccine and disapproval towards a vaccine mandate toward patients. Additionally, our study showed a positive correlation between knowledge of the vaccine and willingness to personally receive it. Lastly, we were able to show that the overwhelming majority of surveyed medical students showed little reluctance to recommend or personally receive it. With much of the public hesitant about receiving the COVID-19 vaccine, it is invaluable to know the attitudes of future physicians that may play a role in largely altering the public’s perspective.

## Supporting information

S1 File (XLSX)
